# Employing Multi-Omics Analyses to Understand Changes during Kidney Development in Perinatal Interleukin-6 Animal Model

**DOI:** 10.3390/cells13191667

**Published:** 2024-10-09

**Authors:** Ganesh Panzade, Tarak Srivastava, Daniel P. Heruth, Mohammad H. Rezaiekhaligh, Jianping Zhou, Zhen Lyu, Mukut Sharma, Trupti Joshi

**Affiliations:** 1 Christopher S. Bond Life Sciences Center, University of Missouri-Columbia, Columbia, MO 65211, USA; gppbioinfo@gmail.com; 2 Section of Nephrology, Children’s Mercy Hospital and University of Missouri at Kansas City, 2401 Gillham Road, Kansas City, MO 64108, USA; tsrivastava@cmh.edu (T.S.); mhrezaiekhaligh@cmh.edu (M.H.R.); 3 Midwest Veterans’ Biomedical Research Foundation (MVBRF), Kansas City, MO 64128, USA; jianping.zhou@va.gov; 4 Children’s Mercy Research Institute, Children’s Mercy Hospital and University of Missouri at Kansas City, Kansas City, MO 64108, USA; dpheruth@cmh.edu; 5 Kansas City VA Medical Center, Kansas City, MO 64128, USA; 6 Department of Electrical Engineering and Computer Science, University of Missouri-Columbia, Columbia, MO 65211, USA; zl7w2@missouri.edu; 7 Department of Biomedical Informatics, Biostatistics and Medical Epidemiology, University of Missouri-Columbia, Columbia, MO 65211, USA; 8 MU Institute for Data Science and Informatics, University of Missouri-Columbia, Columbia, MO 65211, USA

**Keywords:** chronic kidney disease (CKD), interleukin-6 (IL-6), multi-omics, miRNA regulation, epigenetics, co-expression

## Abstract

Chronic kidney disease (CKD) is a leading cause of morbidity and mortality globally. Maternal obesity during pregnancy is linked to systemic inflammation and elevated levels of the pro-inflammatory cytokine interleukin-6 (IL-6). In our previous work, we demonstrated that increased maternal IL-6 during gestation impacts intrauterine development in mice. We hypothesized that IL-6-induced inflammation alters gene expression in the developing fetus. To test this, pregnant mice were administered IL-6 or saline during mid-gestation. Newborn mouse kidneys were analyzed using mRNA-seq, miRNA-seq and whole-genome bisulfite-seq (WGBS). A multi-omics approach was employed to quantify mRNA gene expression, miRNA expression and DNA methylation, using advanced bioinformatics and data integration techniques. Our analysis identified 19 key genes present in multiple omics datasets, regulated by epigenetics and miRNAs. We constructed a regulatory network for these genes, revealing disruptions in pathways such as Mannose type O-glycan biosynthesis, the cell cycle, apoptosis and FoxO signaling. Notably, the Atp7b gene was regulated by DNA methylation and miR-223 targeting, whereas the Man2a1 gene was controlled by DNA methylation affecting energy metabolism. These findings suggest that these genes may play a role in fetal programming, potentially leading to CKD later in life due to gestational inflammation.

## 1. Introduction

There is a growing awareness of the global rise in chronic kidney disease (CKD) and obesity [1,2]. The Developmental Origins of Health and Disease (DOHaD) theory, also known as Barker’s hypothesis, proposes that adverse environmental conditions during development can have lasting effects on health and susceptibility to diseases. Epidemiological studies suggest a connection between maternal obesity and a lower ratio of kidney volume to birth weight in the child, an increased risk of congenital kidney anomalies in offspring, and a higher incidence of CKD in adulthood [3,4,5,6,7,8]. Obesity is recognized as a pro-inflammatory state with elevated inflammatory cytokines, such as interleukin 6 (IL-6) [9,10]. Therefore, the inflammatory environment associated with obesity may impact fetal development, including the kidneys, resulting in an increased vulnerability to CKD later in life [11,12,13,14,15]. To investigate the DOHaD concept regarding increased susceptibility to CKD, we established an animal model through perinatal administration of IL-6.

The perinatal IL-6 animal model was developed based on several key principles: (a) obesity-related systemic inflammation is characterized by heightened levels of pro-inflammatory cytokines, including IL-6 [2,3]; (b) unlike many other inflammatory cytokines that cannot cross the placental barrier, IL-6 can enter the fetal microenvironment [16]; (c) injecting IL-6 during mid-gestation was linked to hypertension in adult offspring [17,18]; (d) pregnant women with obesity exhibit 1.5–2 times higher levels of serum IL-6 compared to non-obese pregnant women [19,20,21,22]. Through the administration of IL-6 during mid-gestation in pregnant mice to transiently increase the serum IL-6 level~3-fold above the baseline for 3 h, simulating maternal systemic inflammation, we observed low birth weight (LBW), reduced kidney weight, accelerated kidney maturation, and JAK-STAT activation in the newborn pups [23]. This suggests that maternal IL-6, representing systemic inflammation, has the potential to influence the in-utero environment and impact fetal developmental programming.

The specific molecular changes contributing to alterations during kidney development from the environmental influence of maternal obesity are not fully understood. Recent progress in high-throughput omics technology allows for a comprehensive analysis of biological samples’ genomic, epigenomic, proteomic, and metabolomic aspects [24]. This multidimensional approach, integrating various omics techniques and bioinformatic tools, empowers us to explore the interconnectedness of biological processes in health and diseases using large datasets [25,26,27]. We conducted a multi-omics analysis, incorporating mRNA sequencing, miRNA sequencing and whole-genome bisulfite sequencing (WGBS), to explore the alterations during kidney development resulting from intrauterine exposure to IL-6 on gene expression in newborn pups. This approach was aimed at identifying key biological processes and networks.

## 2. Materials and Methods

### 2.1. Ethics Statement

All the experiments involving animals were conducted according to the ethical policies and procedures approved by the Institutional Animal Care and Use Committee (IACUC), the Institutional Biosafety Committee/Subcommittee on Research Safety and Security, and the Research and Development Committee at the Kansas City VA Medical Center, Kansas City, MO, USA (approval no. MS002/00637). The study was carried out in compliance with the ARRIVE guidelines and ethical policies approved by the Research and Development Committee at the KCVA Medical Center, Kansas City, MO, USA.

### 2.2. Animals

We conducted studies involving C57BL/6J mice (Jackson Labs, Bar Harbor, ME, USA) following approved protocols from the IACUC, Safety Subcommittee and the R&D Committee at the VA Medical Center in Kansas City, MO, USA. The mice were housed in AAALAC-approved facilities with unrestricted access to food and water, and they were subjected to a 12/12-h light-dark cycle. Our study adhered to the ARRIVE guidelines. Nulliparous mice were mated overnight, and those with a vaginal plug were separated (embryonic day, E0.5). Pregnant mice in the experimental group received intra-peritoneal IL-6 (10 pg/g bw) every other day from E12.5 until the end of gestation, while control pregnant mice received normal saline. After euthanasia, fetal kidneys (E20.5) were dissected from uterine horns and used for isolating total RNA, miRNA, and DNA, or fixed in OCT.

### 2.3. miRNA and mRNA Isolation with Assessment of Quantity and Quality

The kidneys from control and the IL6 samples with 5 replicates each were preserved in RNAlater and subsequently sliced into smaller fragments, and total RNA was extracted separately utilizing the mirVana™ kit (Life Technologies, Carlsbad, CA, USA) designed for both total RNA and small RNAs. To quantify the RNA concentration, the Quant-iT RiboGreen Kit (Catalog # R11490, Thermo Fisher Scientific, Waltham, MA, USA) was employed, and the BioRad Experion system (BioRad, Hercules, CA, USA), along with the RNA Std Sense Chip, was utilized to evaluate RNA quality. Before miRNA and mRNA sequencing, a denaturing polyacrylamide gel was employed to verify the quality of both small RNAs and mRNA.

### 2.4. Library Preparation and Sequencing for miRNA and mRNA

miRNA and mRNA sequencing libraries were prepared in duplicate using the TruSeq small RNA Sample Preparation Kit and the Illumina TruSeq Stranded Total RNA Sample Prep Kit, respectively. To the 5′ and 3′ ends of small RNA and mRNA, RNA adapters were ligated, followed by reverse transcription and polymerase chain reaction PCR to generate a cDNA library. Afterward, the resulting cDNA libraries underwent quality and size checks, purification on a 6% PAGE gel, concentration via ethanol precipitation, and revalidation. The libraries were then appropriately diluted and stored at −20 °C. For the cluster generation, libraries were denatured, spiked with PhiX control, and loaded onto the cBot for clustering on a flow cell. Subsequently, the flow cell contents were sequenced using a HiSeq 1500 in a paired-end (2 × 101) run with the SBS Kit. Real-time analysis and base calling were executed using the HiSeq Control Software 1.4.5.

### 2.5. Methyl-Seq Library Preparation and Sequencing

The University of Missouri (MU) Genomics Technology Core conducted the enzymatic detection of 5-methylcytosines (5mC) and 5-hydroxymethylcytosines (5hmC) through library construction and sequencing. Libraries were constructed according to the New England Biolab NEBNext^®^ Enzymatic Methyl-seq Kit (Catalog #E7120) protocol, involving the Covaris M220 for gDNA fragmentation. Methyl-Seq adaptors were ligated to the fragmented and end-repaired/dA-tailed genomic DNA. A 2-step enzymatic conversion protecting 5mC and 5hmC from deamination was executed, followed by PCR amplification to enrich the libraries. The final cDNA constructs were purified using Axyprep Mag PCR Clean-up beads to select for a 550 bp insert size. For library quantification, we employed the Qubit HS DNA kit, and fragment size analysis was conducted with an Agilent Fragment Analyzer Automated CE system. The libraries were diluted following Illumina’s standard protocol for sequencing on the NovaSeq 6000 (Illumina, San Diego, CA, USA).

### 2.6. Bioinformatics Analysis

#### 2.6.1. Transcriptome and miRNA Expression Analysis

The initial analysis of mRNA sequencing involved assessing the quality of raw reads using fastqc and fastp v.0.22.0 [28]. The quality assessment results were summarized with MultiQC [29]. The reads with a minimum quality score of 20 were retained, and adapter content was trimmed using autodetected parameters, producing filtered reads categorized into paired-end and unpaired files. Genome alignment was performed with HISAT2, followed by the sorting and conversion of the alignment in SAM format to BAM [30]. The featureCount utility from the subread package created a gene count matrix using genomic annotations. Normalized fragment per kilobase per million mapped fragments (FPKM) values were calculated with the RSEQC script FPKM_count.py [31,32].

Differential gene expression (DEGs) analysis was conducted using the R Bioconductor DESeq2 package [33], with the read count matrix as input and low-count rows excluded to enhance statistical robustness. DEGs were identified based on log2FC cutoff criteria |±0.5| and a False Discovery Rate (FDR) ≤ 0.05.

For miRNA-seq data, cutadapt [34] was used for quality filtering, retaining reads with a quality score of 20 and excluding those shorter than 16 nt. The miRge3.0 pipeline processed filtered reads, utilizing the miRBase database for miRNA identification and Bowtie for alignment [35]. The resulting read count matrix was analyzed with the edgeR package to identify differentially expressed miRNAs (DEmiRs), using log2FC |±0| and FDR ≤ 0.05 as criteria.

#### 2.6.2. Whole Genome Bisulfite Sequencing Data Analysis for DNA Methylated Regions (DMRs)

The analysis involved processing bisulfite-seq reads from whole genome sequencing to identify differentially methylated regions (DMRs) in control and IL6 samples, each with 6 replicates. We established a standardized workflow using open-source tools. Quality assessment was performed with fastqc before and after filtering, and summaries were visualized with MultiQC [29]. Reads with a quality score of at least 20 were retained, with filtering for adapter content, PCR duplicates and overrepresented sequences using the fastp tool.

Bismark [36] was used to map filtered reads to a bisulfite-converted reference genome (C to T and G to A), utilizing the latest Mus musculus (Mm39) genome from Gencode [37]. Deduplicated alignments were processed with the Bismark methylation extractor to generate context-based reports and coverage files, calculating methylation percentages as follows,

$$\%\;ofmethylation\left(base\right)=\frac{Methylated\left(Cs\right)}{Methylated\left(Cs\right)+Unmethylated\left(Ts\right)}$$

where the count of methylated cytosine (*Cs*) reads and the unmethylated (*Ts*) reads counts are utilized.

To identify changes in the methylated regions, we used the edgeR bisulfite-seq DMRs package [38], inputting a genome-wide count matrix for differential methylation analysis. Low-count regions (fewer than 4 reads in 50% of samples) were filtered out, and AnnotationDBI was employed to identify nearby transcription start sites (TSS) and their distances to genomic features.

After estimating the total dispersion, the glmFit function was applied to fit the model, and glmLRT was used to identify DMRs based on comparisons. We also analyzed the methylation levels in gene promoter regions, evaluating upstream (2 kb) and downstream (2 kb) regions. The number of altered DMRs was calculated based on criteria such as log2FC |±1| and *p*-value ≤ 0.05.

#### 2.6.3. Integrative Multi-Omics Data Analysis for Drawing Inferences

Connecting multi-omics datasets is essential for extracting meaningful biological insights. We developed a custom R code to link DEGs, DemiRs, and DMRs. To explore the functions of altered miRNAs, we obtained a comprehensive miRNA–target gene interactions dataset from Tarbase v. 8.0, which includes confirmed interactions, next-generation sequencing-supported interactions, and predictions from TargetScan and miRanda [39]. The list of perturbed miRNAs identified through differential expression was matched with this dataset to extract corresponding target genes.

We performed 2-level and 3-level integration analyses of all omics data, cross-referencing genes linked to promoter DMRs with DEGs showing inverse relationships and matching them with DEmiRs. The relationships between DMRs and DEmiRs were considered reciprocal. Statistical significance was determined based on opposite effects in differential expression. We calculated the Pearson correlation coefficient (PCC) between miRNAs and their target genes, retaining interactions with PCC < −0.5 and *p*-value < 0.05. R v4.2 and the ggplot2 package were used for analysis and visualization, with the circlize library employed to create plots summarizing multi-omics connections [40].

#### 2.6.4. Principle Component Analysis, Gene Set Enrichment Analysis (GSEA), and Regulatory Network Analysis

In analyzing multi-omics sequencing datasets, including transcriptomic, miRNAome, and whole-genome bisulfite sequencing, we organized samples based on metadata using principal component analysis (PCA) and K-means clustering. Normalized count data from DESeq2 and edgeR were utilized, with PCA performed using the “pca” command from the PCAtools R package, excluding low-variance features. PCA plots for PC1 and PC2 were generated using the biplot command [41].

Simultaneously, we conducted gene set enrichment analysis (GSEA) to examine functional categories of altered genes in DEGs, DEmiRs, and DMRs, employing the gprofiler2 R package [42]. Significant gene ontology (GO) categories and KEGG, WIKI, and REACTOME pathways were filtered for FDR < 0.05 and at least one gene for statistical over-representation. R’s ggplot2 was used for visualization, and k-means clustering was performed with the factoextra package.

A summary of multi-omics interactions was visualized in Cytoscape v.7 [43], focusing on miRNA–target gene interactions based on anti-correlation calculations. MiRNA-targeted genes were filtered by PCC < −0.1 and *p*-value < 0.1, creating an interaction matrix for analysis. Nodes were further analyzed for over-representation using the ClueGO plugin [44], and highly connected nodes in the miRNA-directed network were identified using the CytoHubba plugin. Figure S1 outlines the workflow and tools used for the analyses.

### 2.7. Immunofluorescence Staining in Newborn Mouse Kidney

We prepared 4-micrometer-thick sections of OCT-embedded kidneys and performed immune staining for podocalyxin to identify glomeruli, following established protocols (23). Antibodies used included rabbit anti-ATP11C (Invitrogen Catalog # PA5-25361) at 1:100, rabbit anti-KLHL15 (Invitrogen Catalog # PA5-86000) at 1:200, rabbit anti-KHDRBS3 (Invitrogen Catalog # PA5-51392) at 1:50, rabbit anti-ARL3 (Invitrogen Catalog # PA5-120440) at 1:100, goat anti-MAN2A1 (Invitrogen Catalog # PA5-18851) at 1:50 and a goat anti-podocalyxin antibody (Catalog # AF1556, R&D Systems, Minneapolis, MN) at 1:500. Secondary antibodies used were Alexa Fluor 488 goat anti-rabbit (Catalog # A21206) and Alexa Fluor 594 donkey anti-goat (Catalog # A32758) at a 1:200 dilution from Invitrogen, Waltham, MA.

Confocal microscopy was conducted on a Zeiss LSM 510 META microscope with an LSM 510 laser module. All the images were acquired at a fixed setting of 20× magnification (in immersion oil) to capture the entire fetal kidney in 2 images for subsequent immunofluorescence quantification. The photomultiplier tube voltage was consistently maintained during image acquisition to ensure comparable relative fluorescence intensities across all the experimental conditions. Fluorescent staining for the protein was quantified by measuring relative fluorescence units (RFU) in the region of interest using open-source Image-J software [45]. Additionally, images were captured and analyzed further using a BZ-X800 Keyence All-in-One fluorescent microscope (Keyence Corporation, Itasca, IL, USA).

## 3. Results

Genes are fundamental units in genomic information, encoding proteins that are essential for processes such as development, normal physiology, and disease conditions. Their regulation involves factors like transcription factors, miRNA molecules, and DNA methylation, influencing both short-term and long-term functions. To explore how these genes and their regulation impact kidney development during maternal inflammation, we performed mRNA sequencing, miRNA sequencing, and WGBS on kidneys from both control and IL-6-exposed fetuses.

### 3.1. Analysis of Transcriptome of Newborn Kidneys from Pups Exposed to Intrauterine Interleukin-6

**Figure 1:**
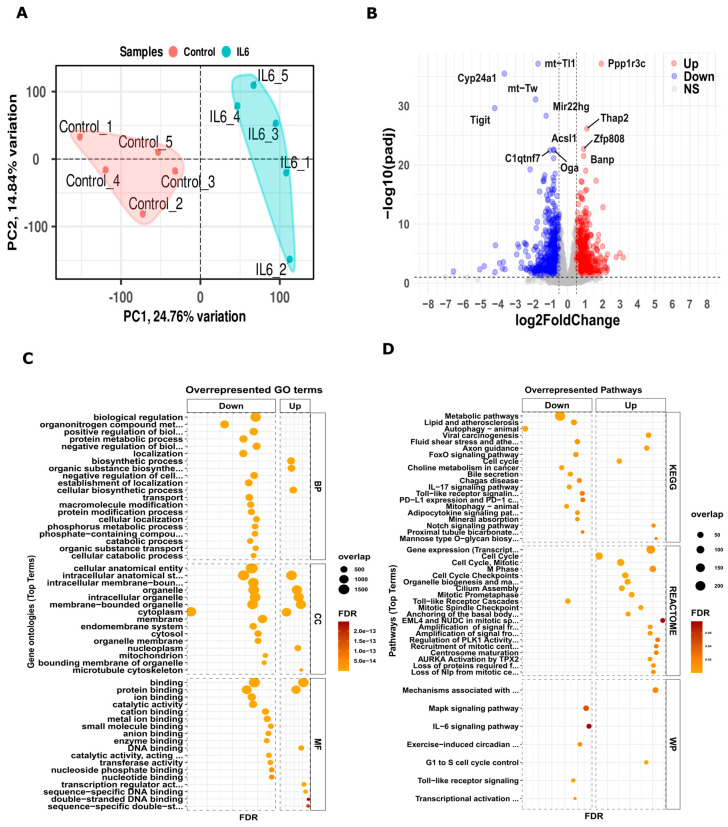
Differentially expressed genes in the transcriptome of kidneys from neonatal pups exposed to IL-6 during development. (**A**) PCA between control and IL-6 samples with the first principal component (PC1) on the *x*-axis and the second (PC2) on the *y*-axis. (**B**) Volcano plot with log2FC on the *x*-axis and *p*-value on the *y*-axis. The red and blue colors denote the altered genes with a one-fold change cutoff of differentially expressed genes. Additionally, (**C**) shows a gene enrichment plot highlighting significantly overrepresented GO terms with an FDR of ≤0.05, while (**D**) focuses on enriched pathways from KEGG, Reactome, and Wiki pathways.

We conducted mRNA-sequencing analysis in five replicates using custom-scripted pipelines that integrate open-source standalone tools (Figure S1). Supplementary Excel S1 Table S1 provides the read numbers for each sample. Using the DESeq2 package to compare IL-6-treated samples to controls, we identified 2361 upregulated and 2518 downregulated genes. These genes met criteria based on a Log2FC value |±0.5| and an FDR value of ≤0.05. Figure 1A illustrates the PCA of the five replicates, while Figure 1B presents a volcano plot highlighting the DEGs.

To understand the biological roles of these DEGs, we conducted an overrepresented gene enrichment analysis. We identified the overrepresented genes using a hypergeometric test and adjusted *p*-values with the Bonferroni–Hochberg correction (BH). Figure 1C presents the predominant terms from the GO categories: biological processes (BP), cellular components (CC), and molecular functions (MF). For a comprehensive view, Supplementary Excel S2 Tables S1 and S2 offer detailed GO results for both upregulated and downregulated genes. Additionally, Figure 1D showcases analyses of the DEGs using KEGG, Reactome, and Wiki pathway databases. These analyses highlighted the genes’ involvement in processes like cell cycle, mitosis, DNA binding, and transcriptional regulation, and various signaling pathways such as Toll-like receptor, IL-17, IL-6, and Notch signaling. Overall, these findings underscore the significance of these DEGs in gestational inflammation during kidney development.

### 3.2. miRNAs and Their Interactions with Target Genes in Newborn Kidneys from Pups Exposed to Intrauterine Interleukin-6

**Figure 2:**
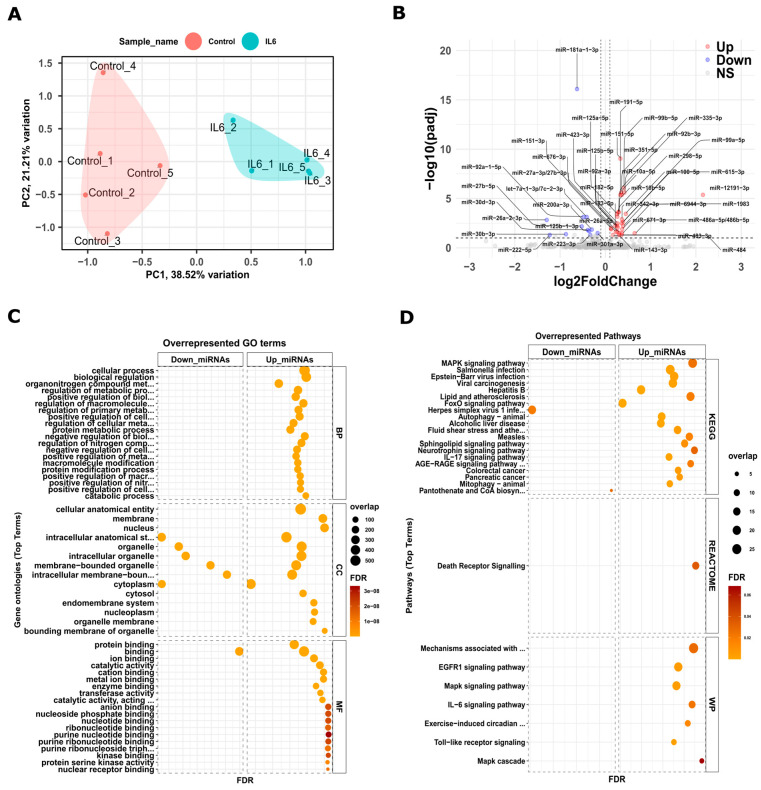
Differentially expressed miRNAs in kidneys from neonatal pups exposed to IL-6 during development. (**A**) PCA between control and IL-6 samples with the first principal component (PC1) on the *x*-axis, whereas the second (PC2) *y*-axis from miRNAs log-transformed counts. (**B**) Volcano plot with log2FC on the *x*-axis and *p*-value on the *y*-axis. The red and blue colors denote the altered genes with a one-fold change cutoff of differentially expressed miRNAs (DEmiRs). The upregulated miRNAs are denoted with red dots and downregulated with blue dots. The gray dots are not significant. Additionally, (**C**) shows a gene enrichment plot highlighting overrepresented GO terms with an FDR of ≤0.05 for downregulated and upregulated miRNAs, while (**D**) focuses on enriched pathways from KEGG, Reactome and Wiki pathways.

We conducted miRNA-sequencing in five independent replicates. Figure S1 outlines the pipeline used to identify key miRNAs and their interactions with target genes. The samples are clustered into control and IL6 categories. Given that miRNAs often exhibit low-level expression with dynamic patterns across different tissues and conditions, the DESeq2 analysis pinpointed 24 upregulated and 13 downregulated DEmiRs. These were determined based on Log2FC values of |±0.5|, coupled with a significant FDR value below 0.05. Figure 2A presents the PCA results for the five replicates, while Figure 2B showcases the volcano plot highlighting the DEmiRs.

To examine the interactions between DEmiRs and their target genes, we focused on the complementary binding within the 3′ UTR of genes, ranging from positions two to eight, both with and without mismatches utilizing the TarBase v8.0 [39]. We kept unique pairs of interactions between miRNA and target genes, regardless of their sequence complementary binding patterns with or without mismatches. We further analyzed target genes inversely expressed by the miRNAs, using GSEA. Figure 2C illustrates the primary terms in GO related to biological processes, cellular components, and molecular functions, while Supplementary Excel S2 Tables S2 and S3 offers a comprehensive view of the GO results for both upregulated and downregulated DEmiRs. Moreover, we evaluated DEmiRs using databases like KEGG, Reactome, and Wiki pathways, as depicted in Figure 2D. Our findings indicate that downregulated target genes of upregulated miRNAs are linked to pathways such as IL-6 signaling, Mapk signaling, death receptor signaling, IL-17 signaling, and FoxO signaling. Conversely, upregulated target genes of downregulated miRNAs are associated with cellular processes, transcriptional regulation, and DNA binding functions. Thus, these miRNA and mRNA interactions play a critical role during kidney development.

### 3.3. Analysis of Whole Genome Bisulfite Sequencing of Newborn Kidneys from Pups Exposed to Intrauterine Interleukin-6

**Figure 3:**
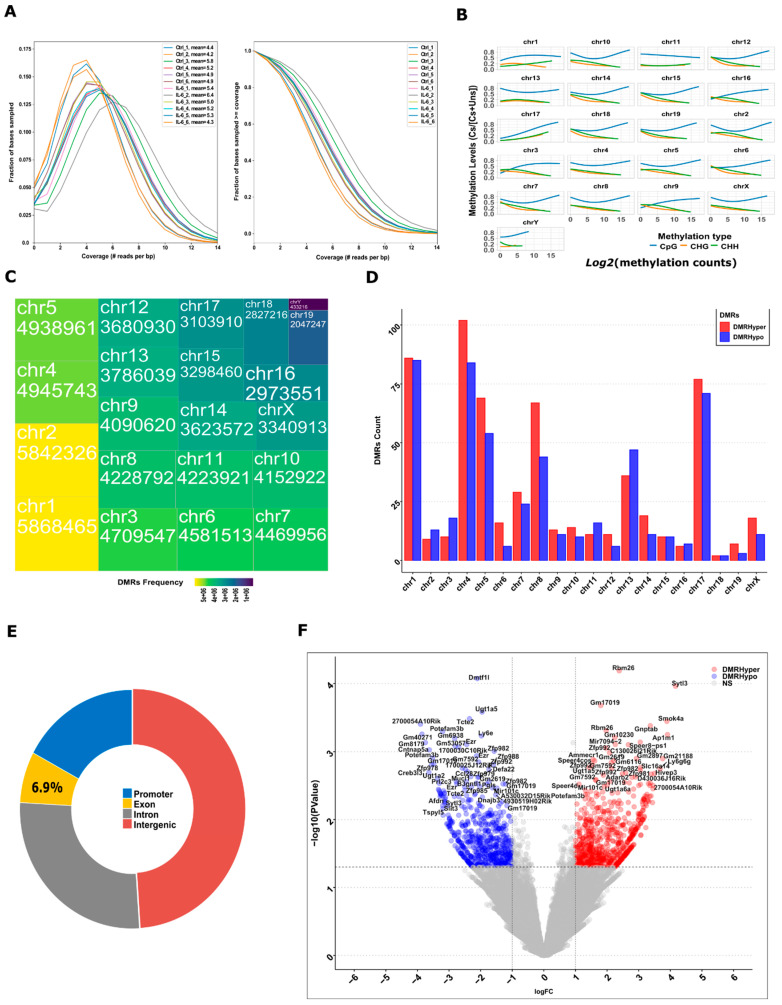
Genome-wide methylation analysis of kidneys from neonatal pups exposed to IL-6 during development. (**A**) Genome-wide coverage of methylated reads per sampled bases. (**B**) Global- and chromosome-wise count of methylated bases in the genome based on the methylation CpG, CHG, and CHH levels ranges between 0.0 and 1.0 and methylation counts. Chromosome-wide coverage of CpG, CHG and CHH methylation. (**C**) Distribution of methylated bases count across the chromosomes in genome. (**D**) Distribution of DMRs based on their methylated categories across the individual mouse chromosome. Distribution of DMRs based on their methylated categories across the chromosome. (**E**) Classification of DMRs based on their chromosomal locations as intergenic and intragenic. Gene enrichment plot for differential methylated genes of promoter and gene body regions. (**F**) Volcano plot of differentially methylated bases in IL-6. The red and blue dots are for hypermethylated and hypomethylated bases, respectively based on 2-fold change and *p*-value < 0.05.

The WGBS experiment involved six replicates to examine DNA methylation patterns. Initially, we processed the reads using the Bismark tool, which produced a coverage file detailing CpG sites, and CHG and CHH sites at the base level. By aligning the reads to the reference genome, we found that most regions had coverage of at least five reads per base, with nearly 20% having seven or more reads per base (Figure 3A). Using the methylation extractor program from the Bismark package, we determined methylation levels by calculating the ratio of methylated *Cs* to the total of methylated *Cs* and unmethylated Ts. This analysis offered a comprehensive genome-wide map of methylation levels. Notably, CpG sites (~74%) displayed the highest methylation levels, aligning with their known role in DNA methylation maintenance. In contrast, CHG (~12%) and CHH (~14%) sites exhibited lower methylation levels, typically associated with DNA modification events rather than maintenance methylation (Figure 3B). To map the distribution of differentially methylated regions across the genome associated with any site like intergenic or genic regions, we identified a total of 81,261,444 methylated regions with coverage exceeding five reads in any replicate from both sample groups within the WGBS data after the initial analysis. Visualization of these methylated regions on mouse chromosomes indicated varied methylation frequencies across different chromosomes, with notable methylation marks observed in most chromosomes, except for chr19 and chrY (Figure 3C).

In the subsequent analysis, we aimed to pinpoint DMRs between the IL-6 and control samples. Using the criteria of a log2FC value of |±1| and a p-value under 0.05, we identified 612 hypermethylated and 533 hypomethylated DMRs. Figure 3D illustrates the distribution of these DMRs across the 20 pairs of chromosomes. Notably, there were no significant alterations in DMRs on chrY. These methylated regions were linked to nearby genes, encompassing the gene’s promoter, protein-coding or noncoding regions, as well as intergenic regions. Across the entire dataset, the majority of methylation marks were located in intergenic regions (48.93%), followed by introns (26.97%), promoter/transcription start site regions (17.26%), and exons (6.94%) (Figure 3F). Specifically, we pinpointed 259 hypermethylated and 226 hypomethylated genes, based on their vicinity to annotated genes. Further, while narrowing down to *Cs* context sites within gene 2kb promoter regions, these numbers reduced to 79 hypermethylated and 212 hypomethylated genes (Figure 3E).

A detailed report was provided for various results of GO terms for these promoter-associated upregulated and downregulated DMRs (Supplementary Excel S2 Tables S5 and S6). To understand the functional implications of these DMRs, we conducted a GSEA focusing on the promoter regions. The analysis revealed overrepresented GO terms related to carbohydrate derivative/small molecule binding, catalytic activity, and the regulation of various biological, cellular and metabolic processes, as well as responses to stimuli. Notably, some genes associated with these DMRs were linked to mitotic cell processes, microtubule cytoskeleton, mitotic spindle, and spindle pole in cellular components. Additionally, there was a notable prevalence of mannose-related molecular functions (Figure S2).

### 3.4. Novel Insights from Integrating 2 Multi-Omics Datasets from Newborn Kidneys of Pups Exposed to Intrauterine Interleukin-6

We employed an integrative approach to identify regulatory patterns involving DNA methylation and miRNA targeting and its impact on gene expression. We systematically analyzed differential patterns using transcriptome, miRNA expression, and WGBS datasets. The overlapping patterns emerged when comparing any two of the available three multi-omics datasets.

In two omics datasets integrative analyses, we discovered a set of 82 hypermethylated regions (DMRs) associated with 27 downregulated genes (DEGs), and 20 hypomethylated regions (DMRs) associated with 12 upregulated genes (DEGs), by integrating DMRs and DEGs (Supplementary Excel S1 Table S2). Among these genes, we found upregulated expression of Bcl2 (B cell leukemia/lymphoma 2) and Smarcal1 (SWI/SNF-related matrix-associated, actin-dependent regulator of chromatin, subfamily a-like 1), which are associated with oligomeganephronia and Schimke immuno-osseous dysplasia with progressive renal failure, respectively, and downregulated expression of Fat1 (FAT atypical cadherin 1), associated with steroid-resistant nephrotic syndrome and progressive renal failure. In the DMRs and DEmiRs omics dataset integration, there was a single association between methylation marks in the promoter region of miR-1902 (Supplementary Excel S1 Table S3).

Likewise, we identified 15 upregulated miRNAs inversely targeting 981 genes, and eight downregulated miRNAs targeting 386 upregulated genes from the DEGs set (Supplementary Excel S1 Table S2). Among the upregulated miRNAs, we observed Cd2ap (CD2-associated protein), Fhl1 (complement component factor H), Sirt1 (sirtuin 1), Wwtr1 (WW domain-containing transcription regulator 1), Fat1, Exoc5 (exocyst complex component 5), Cul3 (cullin 3), and Slc4a4 (solute carrier family 4, member 4), which are associated with end-stage renal disease, membranoproliferative glomerulonephritis, nephrogenic diabetes insipidus, nephronophthisis, steroid-resistant nephrotic syndrome, progressive renal failure, obstructive nephropathy, pseudohypoaldosteronism, and renal tubular acidosis, respectively. The downregulated miRNAs included Arl3 (ADP-ribosylation factor-like 3), Mkks (McKusick-Kaufman syndrome), Ttc8 (tetratricopeptide repeat domain 8), Nphp4 (nephronophthisis 4), Bcl2, and Tulp3 (tubby-like protein 3), which are associated with autosomal recessive polycystic kidney disease, Bardet-Biedl syndrome 6, Bardet-Biedl syndrome 8, nephronophthisis 4, oligomeganephronia, and polycystic kidney disease, respectively.

### 3.5. miRNA-Driven Regulatory Networks in Newborn Kidneys of Pups Exposed to Intrauterine Interleukin-6

**Figure 4:**
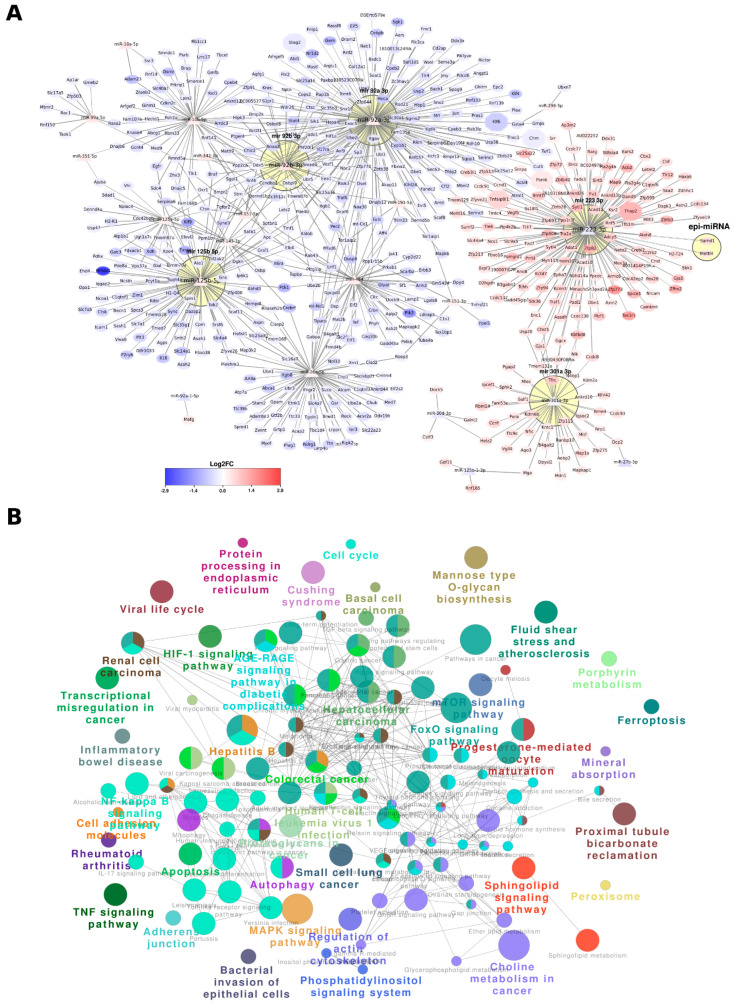
miRNA regulatory network: (**A**) a regulatory network of miRNAs was constructed based on multi-omics analysis with target genes, using anti-correlation. Triangle-shaped nodes represent miRNAs, hexagonal shapes represent genes that overlap with a set of 19 genes, and circles represent other target genes. The miRNA clusters were annotated with yellow circles, and the color scale was based on Log2FC values from differential expressions. (**B**) A miRNA-regulated functional network was created from ClueGo analysis for KEGG and Reactome pathways, with a statistical significance of *p*-value < 0.05. Each cluster within the network was filled with multiple specific colors representing the biological function, with shared functionality represented by multiple colors for the node.

Our investigation harmonized RNA-seq and miRNA-seq datasets, leveraging the reciprocal association between miRNAs and their target genes to establish an extensive miRNA-centric regulatory network. Specifically, we pinpointed 17 of 24 upregulated miRNAs and six of 13 downregulated miRNAs, which collectively targeted 181 upregulated genes and 420 downregulated genes, culminating in the delineation of 764 distinct interactions (Figure 4A, Supplementary Excel S3 Table S1). The interactions were curated through a selection process predicated on an anti-correlation PCC value of <−0.1. Notably, among these interactions, 222 were identified as uniquely falling significantly below the established *p*-value threshold of 0.05. Subsequently, all distinctive interactions were retained in the analysis, irrespective of their statistical significance. Among the 23 miRNAs identified, the top three miRNAs, miR-92a-3p, miR-223-3p and miR-125b-5p, emerged as key regulators, targeting 175, 138 and 90 genes, respectively (Supplementary Excel S3 Tables S2 and S3). Hub nodes analysis revealed that these three miRNAs exhibited the highest number of connections within the regulatory network. Interestingly, while miR-92a-3p and miR-125b-5p shared only 10 common targets, there was no overlap with miR-223-3p, although they each individually targeted a substantial number of genes (175, 90, and 138 genes, respectively) (Figure S3). Furthermore, miR-92a-3p and miR-223 demonstrated evolutionary conservation across multiple species, including humans, mice, rats and zebrafish, and their mode of regulation was conserved in terms of controlling target gene function. In addition, miR-223-3p was also regulated by epi-miRNA Samd1 and Mettl4 (Figure 4).

We constructed a miRNA-regulated functional network using ClueGo analysis for KEGG and Reactome pathways, focusing on those with a statistical significance of *p*-value < 0.05. Within this network, each cluster was color-coded to represent specific biological functions. If nodes shared functionalities, they were depicted with multiple colors (Figure 4B). In total, 39 pathway sets showed connections with the miRNAs’ reciprocal targets (Supplementary Excel S4 Table S1). Notably, as shown in Supplementary Excel S4 Table S1, groups 25–38 were involved in multiple pathways, of which groups 36, 37, and 38 stood out, showing the most extensive representation across various categories in KEGG, Reactome, and WikiPathways. The most overrepresented pathways in these 39 groups were the ErbB signaling pathway, human T-cell leukemia virus 1 infection, PD-L1 expression, and the PD-1 checkpoint pathway in cancer with these three genes (Jun, Kras and Pik3ca), common to these three pathways.

### 3.6. Integrating 3 Multi-Omics Datasets from Newborn Kidneys of Pups Exposed to Intrauterine Interleukin-6

In the integration of all three omics datasets, we performed a promoter-based methylation analysis, which revealed a set of 19 genes with DNA methylation marks in their promoters and targeted by miRNAs. The associations we observed can be categorized into four groups as detailed in Table 1: (a) genes with hypermethylated marks and upregulated DEmiRs, resulting in downregulated DEGs highlighted in red, (b) genes with hypomethylated marks and downregulated DEmiRs, leading to upregulated genes highlighted in blue, (c) genes affected by downregulated DEmiRs and upregulated DEGs with hypermethylated DMRs highlighted in green, and (d) genes influenced by upregulated DEmiRs and downregulated DEGs with hypomethylated DMRs highlighted in orange.

**Figure 5:**
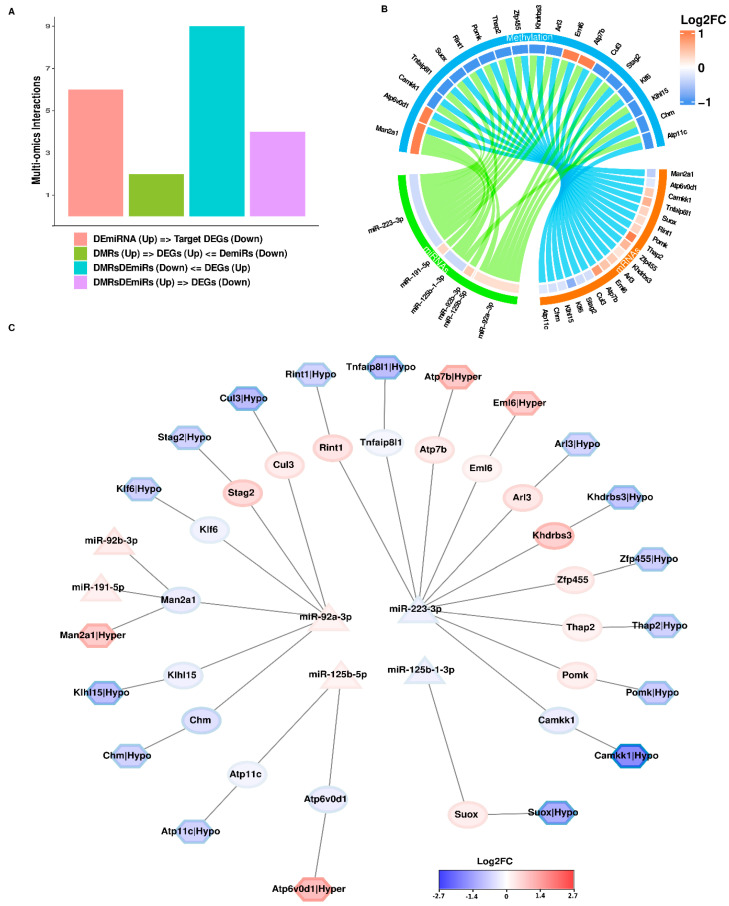
Integration of omics data. Comparative analysis of IL-6 treatment effects from three sets of sequencing data. (**A**) A summary of multi-omics datasets together with differential methylated/expressed interactions between genes and their associated methylation level and miRNAs. The *x*-axis denotes types of regulatory interactions, and the *y*-axis is a calculated percentage of contributing interactions in the total dataset. (**B**) A circular plot for the multi-omics differential methylated expressed genes and miRNAs with log2FC value. (**C**) A regulatory network was constructed of regulatory interactions after the integration of their differential status. The hexagonal shape of nodes denotes the differentially methylated genes (promoter-based), rectangular shape for DEGs, and triangular shape for DemiRs.

These findings highlight a distinct group of genes influenced, not only by DNA methylation, but also by miRNAs. To visualize these interactions and understand their implications in kidney development impacted by maternal inflammation, we created a network diagram (Figure 5A–C and Figure S4).

This network was built to display the regulatory role of DNA methylation and miRNAs for a set of 19 DEGs curated from multi-omics data analysis. It was incorporated with their Log2FC values from the differential analysis of omics data to provide the expression status of genes and miRNAs (Figure 5C). Additionally, Table 2 provides insights into the functional roles of these 19 genes based on existing literature, and a Gonet-based connected network was created to display a crucial role at various checkpoints in different biological functions (Figure S4).

### 3.7. Novel Insights via Protein–Protein Interactions: Co-Expression-Based Functional Network Analysis of 19 Genes Identified in the Multi-Omics Datasets Analyses

**Figure 6:**
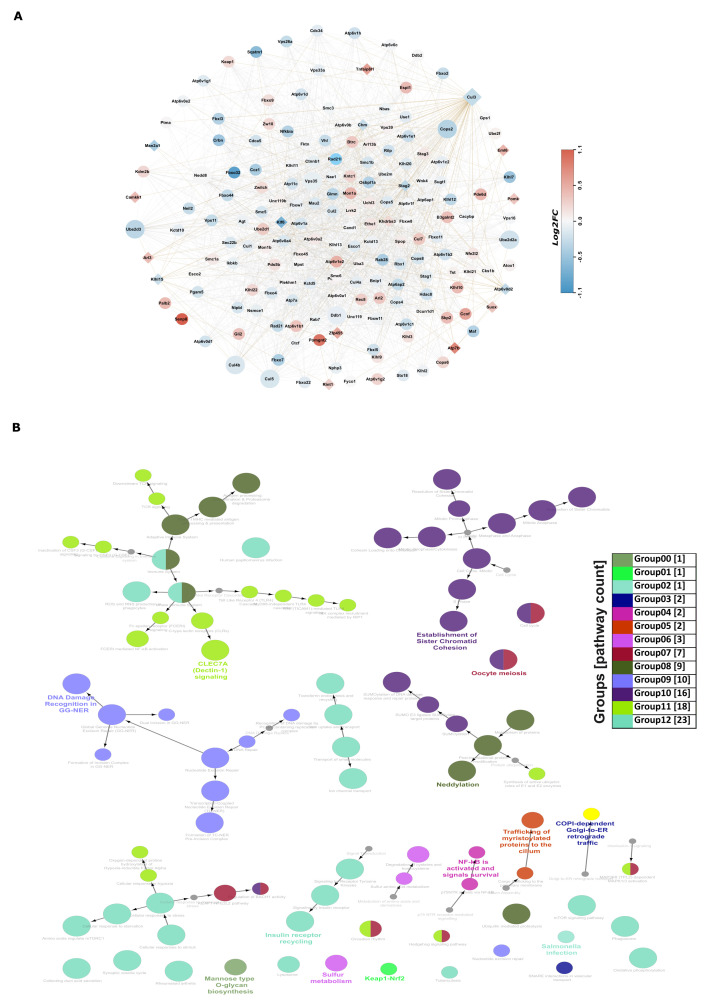
Functional protein–protein network of 19 genes from multi-omics. (**A**) A PPI network was constructed up to 2 layers for the 19 genes, with diamond-shaped nodes denoting the genes that are part of the multi-omics set and circle nodes for PPIs connected to them. The red and blue color scale was based on the differential expression of genes from RNA-seq, while the size of the nodes was based on the number of out-connections. (**B**) A directional functional enrichment network was created for the PPI network using KEGG and Reactome pathways from ClueGO. Shared nodes were divided into half circles, colored, and arrows were used to denote the directions of the function. The size of nodes for a particular function was based on the number of involved genes.

The integration of three omics datasets identified 19 genes. Using the STRING [46] database, we examined the protein–protein interactions (PPI) between these genes, considering connections up to the second layer (Figure 6A).

Of these 19 genes, 18 had connections within the network. Notably, the Thap2 gene only interacted with Klf6 and lacked further connectivity, so was omitted from the network. To understand the PPI network’s functional enrichment, we sorted it into 13 categories based on gene involvement in KEGG, Reactome, and Wiki pathways (Figure 6B). The Cul3 pathway was the most common, appearing in four groups, and interacted directly or indirectly with other genes in the network (Supplementary Excel S4 Table S1). We assessed interactions using a co-expression score of 0.1 (PCC) derived from RNA-seq normalized FPKM values. Additionally, we used a differential expression log2FC scale to color the nodes, emphasizing the highly connected nodes with larger circles. The diamond-shaped nodes represented the 19 identified genes. Overall, we detected 1651 interactions among 182 genes, with positive correlation scores ranging from 0.1 to 0.99 (Supplementary Excel S3 Table S1). Notably, 31% of these interactions had scores above 0.5, while 69% were below 0.5.

### 3.8. Validation of Results Following Integration of Multi-Omics Datasets

**Figure 7:**
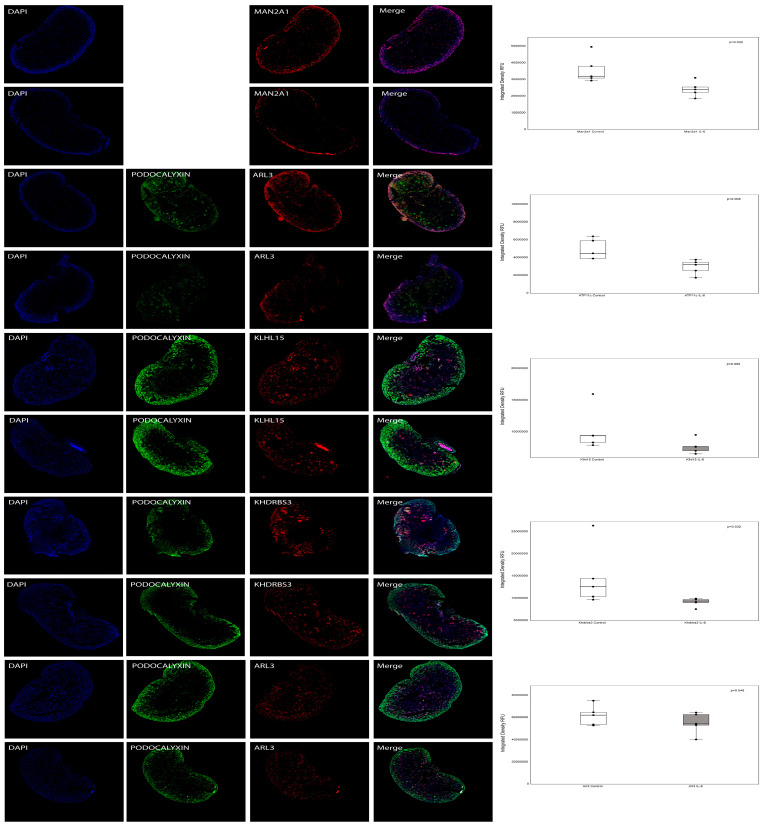
Immunofluorescence microscopy of fetal mouse kidney at birth from pups born to mothers exposed to IL-6, *n* = 5 or saline (control, *n* = 5). Immunofluorescence staining for staining results for Man2a1 (Mannosidase 2 alpha 1), Klhl15 (Kelch-like 15), Khdrbs3 (KH domain containing RNA binding signal transduction associated 3), Atp11c (ATPase class VI type 11C), and ADP-ribosylation factor-like 3 (Arl3) are shown in the following panel. Glomeruli are stained for podocalyxin (green fluorescence), while Man2a1, Klhl15, Khdrbs3, Atp11c, and Arl3 are stained with red fluorescence. The podocalyxin staining for Man2a1 was not performed because both the primary antibodies were from goat. All confocal images were taken at fixed acquisition settings. Boxplots show fluorescence intensity analysis with median and box representing interquartile range (25th to 75th percentile), and 95% of data were within limits of whiskers. Wilcoxon–Mann–Whitney tests were conducted to assess differences between the two groups.

We validated some of the genes identified in the multi-omics datasets in newborn kidneys from pups exposed to intrauterine IL-6. Figure 7 displays the immunofluorescence staining results for Man2a1 (Mannosidase 2 alpha 1), Klhl15 (Kelch-like 15), Khdrbs3 (KH domain containing RNA binding signal transduction associated 3), Atp11c (ATPase class VI type 11C), and ADP-ribosylation factor-like 3 (Arl3) in kidneys exposed to either saline or IL-6 across five replicates.

Table 3 provides the quantified results for RFUs on confocal microscopy, including *p*-values, obtained using the open-source Image-J software. We used Wilcoxon–Mann–Whitney tests to assess the differences between the two groups. In line with the transcriptome data, the expression of Man2a1 and Atp11c showed a significant decrease, while Klhl15 exhibited an almost significant decrease. In contrast, Arl3 did not show a significant change, and although Khdrbs3 had a significant alteration at the protein expression, it was in the opposite direction to the gene expression.

## 4. Discussion

Barker’s hypothesis (or DOHaD concept) suggests that environmental factors during development can increase an individual’s risk of disease later in life. Epidemiological evidence supports that maternal obesity can lead to smaller kidneys, a higher likelihood of congenital kidney issues in offspring and an increased risk of CKD in adulthood [5,6,7,8,9,10]. To study the impact of maternal obesity on CKD risk, we created an animal model where IL-6 was administered during the perinatal period to pregnant dams, resulting in offspring with LBW and smaller kidneys [23]. Unlike genetic mutations, the effects of the prenatal environment on development are subtle, involving changes like DNA methylation, histone modifications, and alterations in mRNA and miRNA expression. To delve deeper into how maternal obesity affects kidney development, we performed sequencing studies for the mRNA, miRNA, and WGBS of these fetal kidneys.

Initially, we carried out a standard single omics analysis. From our mRNA sequencing dataset, we observed 2361 genes that were upregulated and 2518 genes that were downregulated. MicroRNAs (miRNA, ~22nt) regulate protein synthesis through the post-transcriptional inhibition/degradation of mRNA affecting development, proliferation, differentiation, apoptosis, and stress responses [47,48,49,50]. Additionally, our miRNA sequencing revealed 24 upregulated and 13 downregulated miRNAs associated with altered target genes. Figure 1 and Figure 2 illustrate the pathways and biological processes we identified from these DEGs’ and miRNAs’ interactions. These pathways predominantly relate to inflammation, indicating networks influenced by both the DEGs and miRNAs in an inflammatory maternal environment. Although these results offer support and valuable insights, independently they would perhaps lack sufficient guidance for shaping future research directions. This is where having another multi-omics dataset, namely WGBS, helped connect the findings better.

Epigenetic modifications, which lead to changes in phenotype without altering the DNA sequence, can impact kidney development and disease progression [51]. In our animal model, injecting IL-6 into pregnant mice notably increased the presence of 5-methyl-deoxycytosine, but not the 5-hydroxymethyl-deoxycytosine levels in fetal kidney DNA, as observed through LC-MS. This suggests that epigenetic alterations due to an abnormal prenatal environment influence genomic changes during development in our animal model [23]. In our WGBS, we found a total of 81,261,444 DNA methylated regions, which had a coverage of over five reads in any replication across both sample groups. Approximately 74% of these DNA methylated regions were found over CpG sites, whereas 12% and 14% were located over CHG and CHH sites, respectively. In terms of location, these DNA methylated regions predominantly existed in intergenic regions (48.93%), followed by introns (26.97%), promoter and transcription start sites (17.26%) and exons (6.94%), as illustrated in Figure 3. Specifically, our analysis revealed 612 hypermethylated and 533 hypomethylated DNA methylated regions. Interestingly, our findings align with a study involving 237 newborns. This study indicated that approximately 75% of variably methylated regions across roughly 1400 genetic loci could be attributed to factors related to the prenatal environment, such as maternal smoking, depression, or BMI. In contrast, genetic factors accounted for only 25% of these variations [52]. Furthermore, when visualizing these DNA methylated regions on mouse chromosomes, we observed varying methylation frequencies across most chromosomes, except for chr19 and chrY, as depicted in Figure 3C. The disparity in DNA methylation regions’ patterns between the X and Y chromosomes indicates the necessity for more in-depth studies to understand why men often exhibit a higher susceptibility to CKD than women.

The integration of separate ‘-omics’ datasets, known as multi-omics integration, is gaining popularity in contemporary scientific research. Using a multi-omics approach has become common for gathering data from different platforms such as mRNA, miRNA, WGBS and genome-wide association studies GWAS. These varied datasets provide detailed insights into both transcriptomic and genome-wide molecular levels. Thanks to significant advancements in sequencing technologies over the past 20 years, producing data has become more cost-effective. This cost reduction has led to a vast increase in available data for bioinformatics analysis, covering studies from humans to model organisms across various omics experiments [26,53]. Initially, our standard approach with single omics had shown experimental relevance, but lacked specificity for generating specific hypotheses for future studies. To address this, we first combined the data from two groups. The integration revealed associations between DNA methylation regions and 27 downregulated genes, 12 upregulated genes, and the downregulation of miR-1902 (Supplementary Excel S1 Table S3). MiRNAs play a crucial role during development by amplifying networks via acting on multiple mRNAs simultaneously. Among them, the three prominent miRNAs that stood out in our study were miR-92a-3p, miR-223-3p, and miR-125b-5p. They target 175, 138, and 90 genes, respectively, with little overlap among them, as illustrated in Figure 4 and Supplementary Excel Table S2. Analyzing these networks highlighted 39 distinct pathways, detailed in Supplementary Excel S4 Table S1. By integrating two omics groups, we refined our focus on specific genes, pathways and networks.

Finally, in our study we combined three types of omics datasets, mRNA, miRNA, and WGBS-sequencing, allowing us to analyze and interpret data from these varied sources comprehensively and to better connect our findings to each other. Although it is common to integrate two omics datasets, merging three is less typical. Our approach’s strength lies in utilizing expression-based differential and correlation methods to refine the data based on statistical significance. To fully understand gene expression, we focused on methylation analysis in gene promoters, covering both the 3′ and 5′ ends. Changes in methylation at these promoter regions can notably affect gene expression. Such methylation alterations can impact either transcription or alternative splicing. Although the majority of methylation sites are seen in the intergenic areas, exploring intergenic regions beyond typical promoter areas is challenging, given enhancers located 5–10 k upstream of genes lack comprehensive annotations, making it difficult to associate them with specific genes. We focused on the CpG areas because DNA methylation is predominantly studied in the CpG context, given that 80% of methylation events occur at CpG sites.

Using this method, we identified a prominent group of 19 genes influenced by both epigenetic changes and miRNAs, as depicted in Figure 5. After consolidating all three omics datasets, we detailed the roles of these 19 genes in biological systems in Table 2. Among these, 11 genes maintained a consistent relationship: changes in gene expression correlated with both DNA methylation and miRNA expression. However, for the remaining eight genes, whereas the miRNA expression pattern remained consistent, the DNA methylation did not, as illustrated in Table 1. The traditional view that DNA methylation merely inhibits gene expression persists. Yet, advanced DNA methylation analysis methods like WGBS, made possible by high-throughput sequencing, have unveiled a more intricate role for DNA methylation. Though it can silence genes, that is just one aspect of its effects [54]. Evidence indicates that DNA methylation also plays roles in gene activation [55], splicing regulation [56], nucleosome positioning [57,58,59], and transcription factor recruitment [60]. These varied functions suggest that DNA methylation functions more like a cellular epigenetic memory [61,62,63].

The integrated approach resulted in a confident set of 19 genes. We experimentally validated five of these genes, as shown in Figure 5. Specifically, Man2a1 and Atp11c were significantly downregulated, whereas Klhl15 was nearly significantly downregulated (*p*-value of 0.095). In contrast, there was no observable change in Arl3 (Table 3). Notably, Khdrbs3 exhibited a significant change; however, its gene and protein expressions moved in opposite directions. Our experimental validation instills confidence in our multi-omics integrated data for future studies.

Based on these findings, our subsequent studies will emphasize three miRNAs and their consistent targets across analyses: miR-125b-5p, associated with Atp6v0d1 and Atp11c; miR-223-3p, associated with Camkk1; Tnfaip8l1; Rint1; Pomk; Thap2; Zfp455; Khdrbs3; Arl3; Eml6; Atp7b and miR-92a-3p, associated with Man2a1; Cul3; Stag2; Klf6; Klhl15; and Chm.

In our animal model, administering IL-6 during mid-gestation in pregnant mice resulted in LBW, decreased kidney weight, and accelerated kidney maturation [23]. Our focus was on the reduced nephron count and LBW observed in this model. In humans, the number of nephrons in each kidney varies widely, ranging from 210,332 to 1,825,380 [64]. Notably, although the nephron count remains consistent across races, it tends to be lower in females and premature infants. The primary factor influencing nephron count is birth weight. Specifically, for every kilogram increase in birth weight, there are an additional 257,426 nephrons per kidney [65]. Numerous studies have highlighted that LBW, defined as less than 2500 g, has been associated with the progression of various health conditions in both adults and children, including CKD, end-stage kidney disease, hypertension, albuminuria, and cardiovascular disease. Meta-analyses from these studies reveal substantial risks associated with LBW, including an 81% increased risk for albuminuria, 58% for end-stage kidney diseases (ESKD), 79% for CKD, and 21% for hypertension [66,67,68]. Understanding the alterations in kidney development leading to reduced nephron count and lower birth weight is crucial. Such insights will help elucidate the increased susceptibility to CKD later in life. Thus, based on our multi-omics analyses, we are particularly interested in exploring pathways, such as mannose type O-glycan biosynthesis involving Pomk and Man2a1; cell cycle and apoptosis related to Man2a1, Cul3, Stag2, Klf6, Klhl15, and Chm [69,70,71,72], and Golgi function, encompassing Man2a1, Cul3, Arl3, and Rint1 [73]; and spindle and microtubule formation in cells, involving Stag2, Cul3, Arl3, and Eml6 in our animal model.

## 5. Conclusions

In conclusion, when compared to genetic mutations, the effects of the in-utero environment on an organism’s development are more nuanced, influencing DNA methylation, histone modifications and the expression levels of mRNA and miRNA. Our distinct multi-omics integration approach provided advantages beyond traditional experimental methods and single omics studies. By concurrently analyzing diverse data types, we deepened our insights into kidney development and its underlying biological mechanisms. This integrated method uncovered complex interactions between datasets, offering insights that single or dual omics approaches might miss. Our primary goal was to gain a comprehensive understanding of kidney development through detailed multi-data analysis, which would allow us to delve into connections across various omics datasets, crucial for understanding developmental changes. We recognize that epigenetic reprogramming adjusts to environmental conditions primarily to maintain genome integrity. Nevertheless, under adverse conditions, this reprogramming can modify methylation patterns, a process referred to as fetal programming. Such changes may heighten susceptibility to CKD later in life, a subject we plan to further investigate in future studies.

## Figures and Tables

**Table 1:** The table shows a list of 19 DEGs identified following the integration of multi-omics datasets with their associated methylated state in the promoter region and their associated targeting DemiRs. The downregulated genes with hypermethylated DMRs and upregulated DEmiRs are shown in the red category, upregulated genes with hypomethylated DMRs and downregulated DEmiRs are shown in the blue category, upregulated genes with hypermethylated DMRs and downregulated DEmiRs are shown in the green category and downregulated genes with hypomethylated DMRs and upregulated DEmiRs are shown in the orange category.

Category		DMRs		DEGs		DEmiRs		
	Gene_ID	Log2FC	*p*-Value	Log2FC	FDR	miRNA_id	Log2FC	FDR
	Man2a1	1.224	0.002	−0.452	1.68933 × 10^−8^	miR-92b-3p	0.421	0.002
	Man2a1	1.224	0.002	−0.452	1.68933 × 10^−8^	miR-191-5p	0.33	0.002
	Man2a1	1.224	0.002	−0.452	1.68933 × 10^−8^	miR-92a-3p	0.265	0.014
	Atp6v0d1	1.548	0.038	−0.214	0.031	miR-125b-5p	0.266	0.015
	Camkk1	−2.714	2.23904 × 10^−6^	0.42	0.047	miR-223-3p	−0.333	0.026
	Tnfaip8l1	−1.543	0.004	0.623	0.001	miR-223-3p	−0.333	0.026
	Suox	−1.929	0.004	0.353	0.048	miR-125b-1-3p	−0.36	0.031
	Rint1	−1.046	0.008	0.29	0.01	miR-223-3p	−0.333	0.026
	Pomk	−1.217	0.016	0.355	0.036	miR-223-3p	−0.333	0.026
	Thap2	−1.111	0.03	1.105	7.02311× 10^−27^	miR-223-3p	−0.333	0.026
	Zfp455	−1.154	0.034	0.575	0.023	miR-223-3p	−0.333	0.026
	Khdrbs3	−1.32	0.034	0.221	0.037	miR-223-3p	−0.333	0.026
	Arl3	−1.018	0.036	0.409	0	miR-223-3p	−0.333	0.026
	Eml6	1.171	0.014	0.469	0.001	miR-223-3p	−0.333	0.026
	Atp7b	1.294	0.017	0.806	0.005	miR-223-3p	−0.333	0.026
	Cul3	−1.59	0.003	−0.304	0.001	miR-92a-3p	0.265	0.014
	Stag2	−1.052	0.008	−0.347	0.001	miR-92a-3p	0.265	0.014
	Klf6	−1.083	0.018	−0.733	8.18305 × 10^−5^	miR-92a-3p	0.265	0.014
	Klhl15	−1.493	0.02	−0.263	0.04	miR-92a-3p	0.265	0.014
	Chm	−1.067	0.021	−0.287	0.002	miR-92a-3p	0.265	0.014
	Atp11c	−1.082	0.045	−0.215	0.024	miR-125b-5p	0.266	0.015

**Table 2:** The table shows the functional role of the 19 genes identified following the integration of multi-omics datasets from BP, CC, MF, phenotype, or associated disease, and their references from literature.

Genes	Description	BP, CC, MF	PHENOTYPE or Associated Disease	Reference
Arl3	ADP-ribosylation factor-like 3,	Mitotic cytokinesis, kidney development, cell cycle	Autosomal recessive polycystic kidney disease	PubMed ID: 16565502
Atp11c	ATPase, class VI, type 11C	Pre-B cell differentiation, phospholipid translocation	Abnormal B cell differentiation	PubMed ID: 21423173; 21873635;
Atp6v0d1	ATPase, H+ transporting, lysosomal V0 subunit D1	Vacuolar transport	Abnormal kidney morphology	PubMed ID: 21873635
Atp7b	ATPase, Cu++ transporting, beta polypeptide	Copper ion transport, intracellular copper ion homeostasis	Wilson disease, decreased body weight	PubMed ID: 11237756; 21873635; 6863890
Camkk1	Calcium/calmodulin-dependent protein kinase kinase 1, alpha	Intracellular signal transduction, positive regulation of protein kinase activity	Reproductive system phenotype	PubMed ID: 21873635; 17015467
Chm	Choroidermia (RAB escort protein 1)	Vesicle-mediated transport, protein geranylgeranylation	Choroideremia, abnormal visceral yolk sac morphology	PubMed ID: 21873635; 15242790
Cul3	Cullin 3	Negative regulation of transcription by RNA polymerase II, protein polyubiquitination, mitotic cell cycle, kidney development	Pseudo-hypoaldosteronism, abnormal cell cycle, abnormal gastrulation	PubMed ID: 17339333; 27708159; 25250572; 10500095
Eml6	Echinoderm microtubule-associated protein like 6	Microtubule binding	Not Applicable	PubMed ID: 21873635
Khdrbs3	KH domain containing, RNA binding, signal transduction associated 3	Regulation of mRNA splicing, via spliceosome, mRNA binding	Reproductive system phenotype	PubMed ID: 22196734; 21873635; 23637638
Klf6	Kruppel-like factor 6	Cytokine-mediated signaling pathway, positive regulation of connective tissue replacement, DNA-binding transcription factor activity, RNA polymerase II-specific	Decreased body size and cell proliferation	PubMed ID: 10880228; 22820290; 21873635
Klhl15	Kelch-like 15	Nuclear protein quality control by the ubiquitin–proteasome system	Not Applicable	PubMed ID: 21873635;
Man2a1	Mannosidase 2, alpha 1	In utero embryonic development, N-glycan processing, extracellular space	Abnormal renal glomerulus morphology, abnormal renal/urinary system physiology	PubMed ID: 21873635; 16754854; 11158608; 19710420
Pomk	Protein-O-mannose kinase	Protein O-linked glycosylation, brain development, learning or memory, endoplasmic reticulum membrane	Hydrocephalus	PubMed ID: 21873635; 23929950
Rint1	RAD50 interactor 1	Retrograde vesicle-mediated transport, Golgi to endoplasmic reticulum, cell cycle	Abnormal embryo development	PubMed ID: 21873635; 17470549
Stag2	Stromal antigen 2	Sister chromatid cohesion, stem cell population maintenance, chromatin, chromatin binding	Abnormal mitosis, decreased B cell number, decreased body weight, decreased cell proliferation, decreased embryo size, decreased fibroblast proliferation	PubMed ID: 21873635; 20720539; 23920377;22780989; 32783938; 32249213
Suox	Sulfite oxidase	Sulfur compound metabolic process, mitochondrion, molybdopterin cofactor binding	Not Applicable	PubMed ID: 21873635; 14651853; 18614015
Thap2	THAP domain containing apoptosis-associated protein 2	Nucleus	Not Applicable	PubMed ID: 21873635
Tnfaip8l1	Tumor necrosis factor, alpha-induced protein 8-like 1	Negative regulation of TOR signaling, regulation of apoptotic process, protein binding	Abnormal urinary bladder morphology, abnormal kidney morphology	PubMed ID: 21600655; 21873635; 21600655
Zfp455	Zinc finger protein 455	RNA polymerase II cis-regulatory region sequence-specific DNA binding	Not Applicable	PubMed ID: 21873635

**Table 3:** The table shows the median and interquartile range for immunofluorescence staining for the protein on the confocal microscopy as RFUs in the region of interest using Image-J software. All the images were acquired at a fixed setting during image acquisition to ensure comparable relative fluorescence intensities across all the experimental conditions. Wilcoxon–Mann–Whitney tests were used to assess the differences between the two groups (*p* < 0.05 was considered to be significant).

Gene	N	Control Kidney	IL-6 Kidney	*p*-Value
Man2a1	5	3,207,680 (3,099,669, 3,810,075)	2,415,094 (2,237,882, 2,553,436)	0.032
Klhl15	5	9,419,328 (8,376,423, 9,450,573)	7,051,472 (6,556,358, 7,677,648)	0.095
Khdrbs3	5	12,632,619 (10,391,560, 4,424,368)	9,188,871 (9,078,808, 9,727,293)	0.032
Atp11c	5	4,517,416 (3,907,210, 5,916,229)	3220225 (2574163, 3491679)	0.008
Arl3	5	62,159,247 (53,843,077, 64,752,901)	54,465,211 (53,381,556, 62,712,896)	0.548

## Data Availability

Sequencing data files are available on NCBI Sequence Read Archive under the submission number SUB14287235 and BioProject ID: PRJNA1090053.

## Supplementary Materials

The following supporting information can be downloaded at https://www.mdpi.com/article/10.3390/cells13191667/s1; Figure S1: a systematic of the bioinformatics methods to analyze the multi-omics datasets; Figure S2: gene enrichment results for the 19 gene sets from omics analysis; Figure S3: three miRNAs and their target genes’ overlap from anti-correlation analysis; Figure S4: GOnet database GO network of 19 genes in various functional categories; Figure S5: GO Cluego enrichment for the co-expressed PPIs; Supplementary Excel S1: Table S1: Sequencing data read statistics, Table S2: Differential counts of methylated regions, Genes, miRNAs from WGB-seq, mRNA-seq and miRNA-seq, Table S3: The integration of differentially regulated genes with their reciprocal hypermethylated and hypomethylated DNA methylation region (DMR) revealed 82 hypermethylated region associated with 27 down regulated genes and 20 hypomethylated region associated with 12 up regulated genes as shown in the table. There was a single miRNA that was associated with hypermethylated region; Supplementary Excel S2: Table S1: Upregulated differential expressed genes (DEGs) geneset enrichment analysis, Table S2: Downregulated differential expressed genes (DEGs) geneset enrichment analysis, Table S3: Upregulated differential expressed miRNA (DEmiRs) targets geneset enrichment analysis, Table S4: Downregulated differential expressed miRNA (DEmiRs) targets geneset enrichment analysis, Table S5: Upregulated differential methylated regions (DMRs) associated with promoter region geneset enrichment analysis, Table S6: Downregulated differential methylated regions (DMRs) associated with promoter region geneset enrichment analysis, Table S7: Gonet enrichment network based on 19 genes set from multi-omics integration, Table S8: Cluego enrichment analysis for 19 genes and it’s PPI network genes, Table S9: Cluego enrichment analysis for differential expressed miRNAs from regulatory network target genes; Supplementary Excel S3: Table S1: Coexpression based protein protein interaction (PPI) network for 19 genes set from multi-omics integration, Table S2: Differential expressed miRNAs (DEmiRs) and their anit-correlated targets through prediction, Table S3: miRNAs and their target genes with log2FC and PCC scores; Supplementary Excel S4: Table S1: miRNA-based regulatory network-based target genes with GO terms and involved genes with their respective color groups.
