# Supplementary material for: Employing Multi-Omics Analyses to Understand Changes during Kidney Development in Perinatal Interleukin-6 Animal Model
Source: Cells. 2024 Oct 9;13(19):1667. doi: 10.3390/cells13191667 (PMC11476440; doi:10.3390/cells13191667)
Supplement: Supplementary file 1 [file cells-13-01667-s001.zip › Supplementary Figures.pdf]

Supplementary Figures

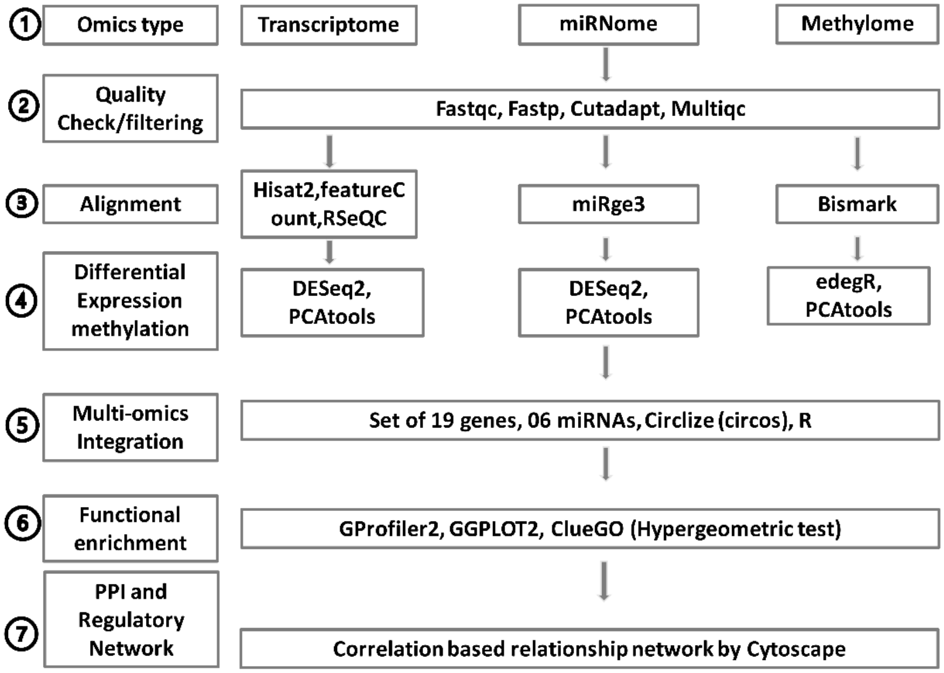

Figure S1: A systematic of the bioinformatics methods to analyze the omics datasets.

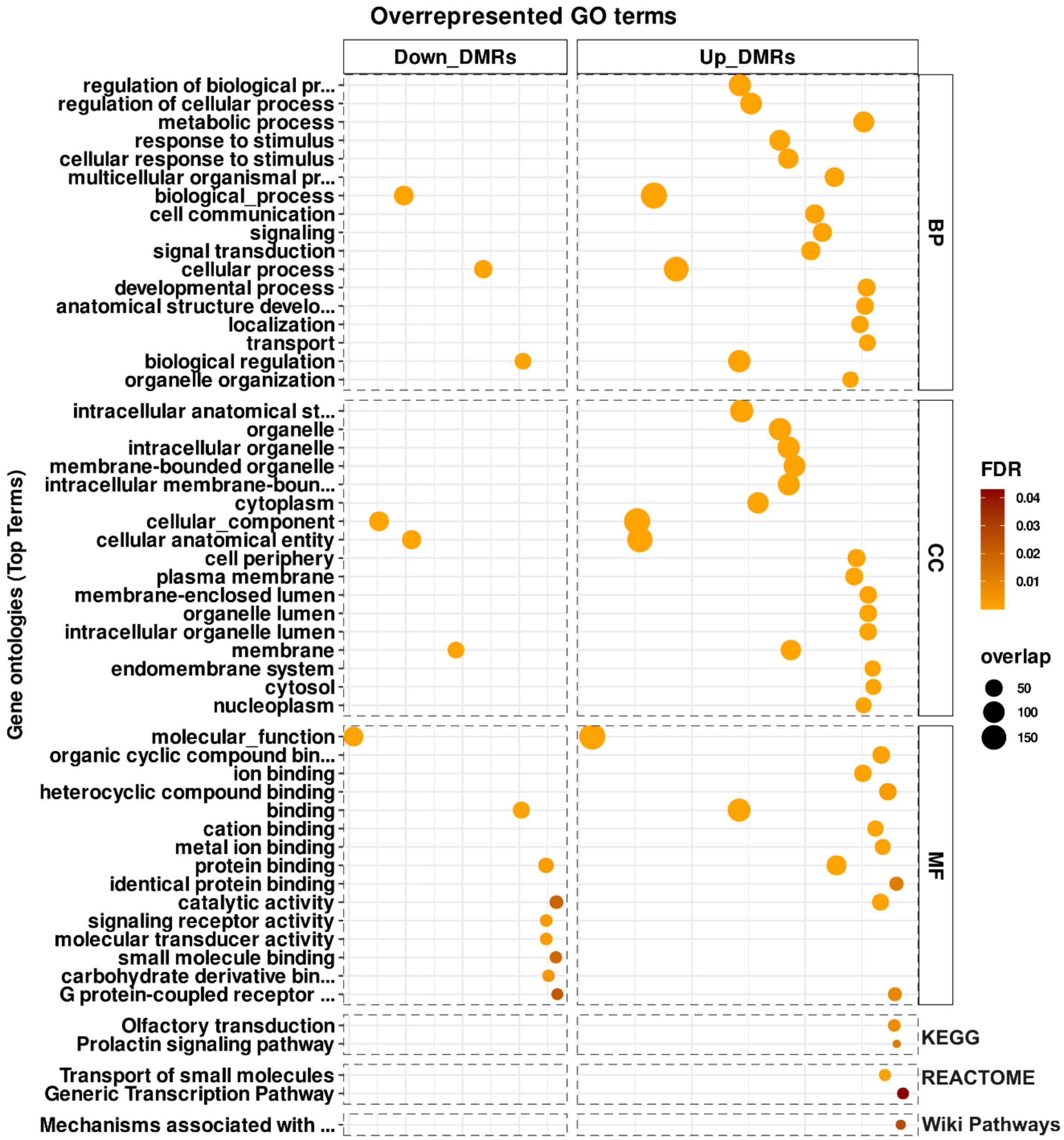

Figure S2: Gene enrichment results for the nineteen gene set from omics analysis.

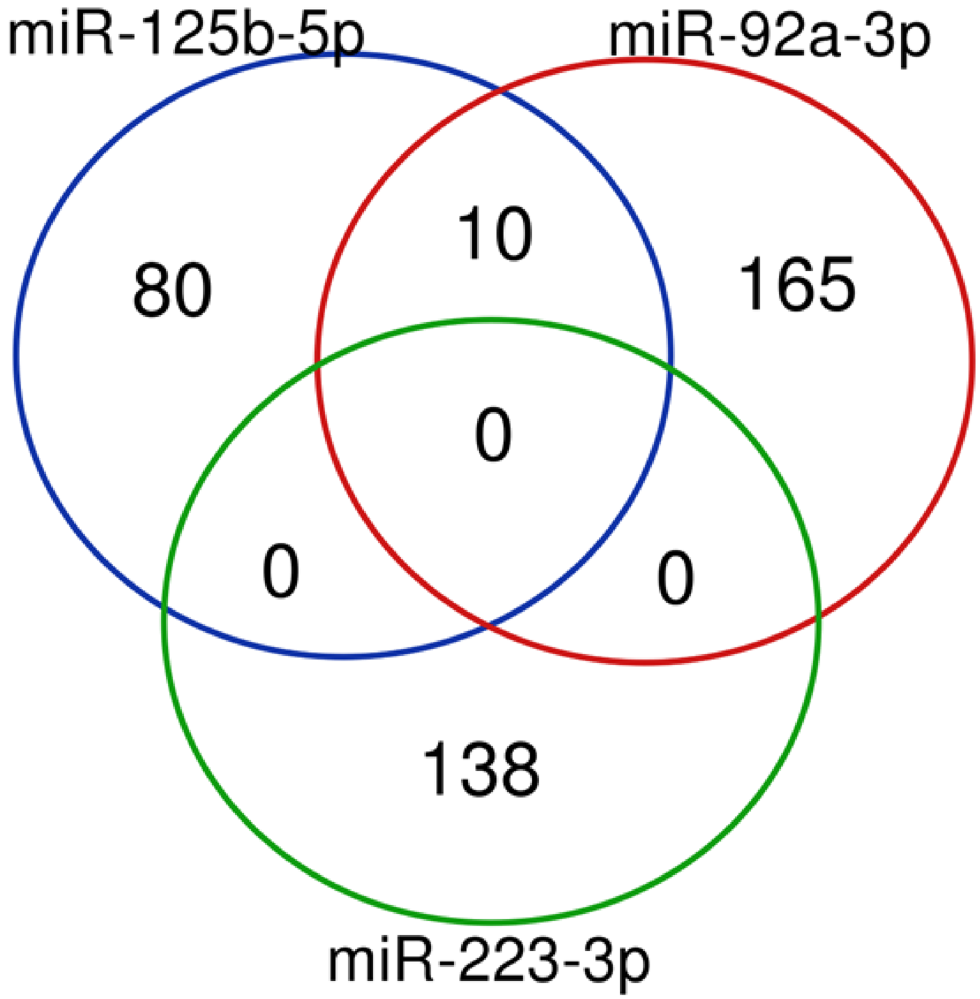

Tmf1, Bmpr2, Pde4b, Sgpl1, Aldob, Lats2, Dennd1b, Tgfbr2, Rnf4 and Strn are the genes shared commonly between miR-92a-3p and miR-125b-5p.

Figure S3: Three miRNAs and their target genes overlap from anti-correlation analysis.

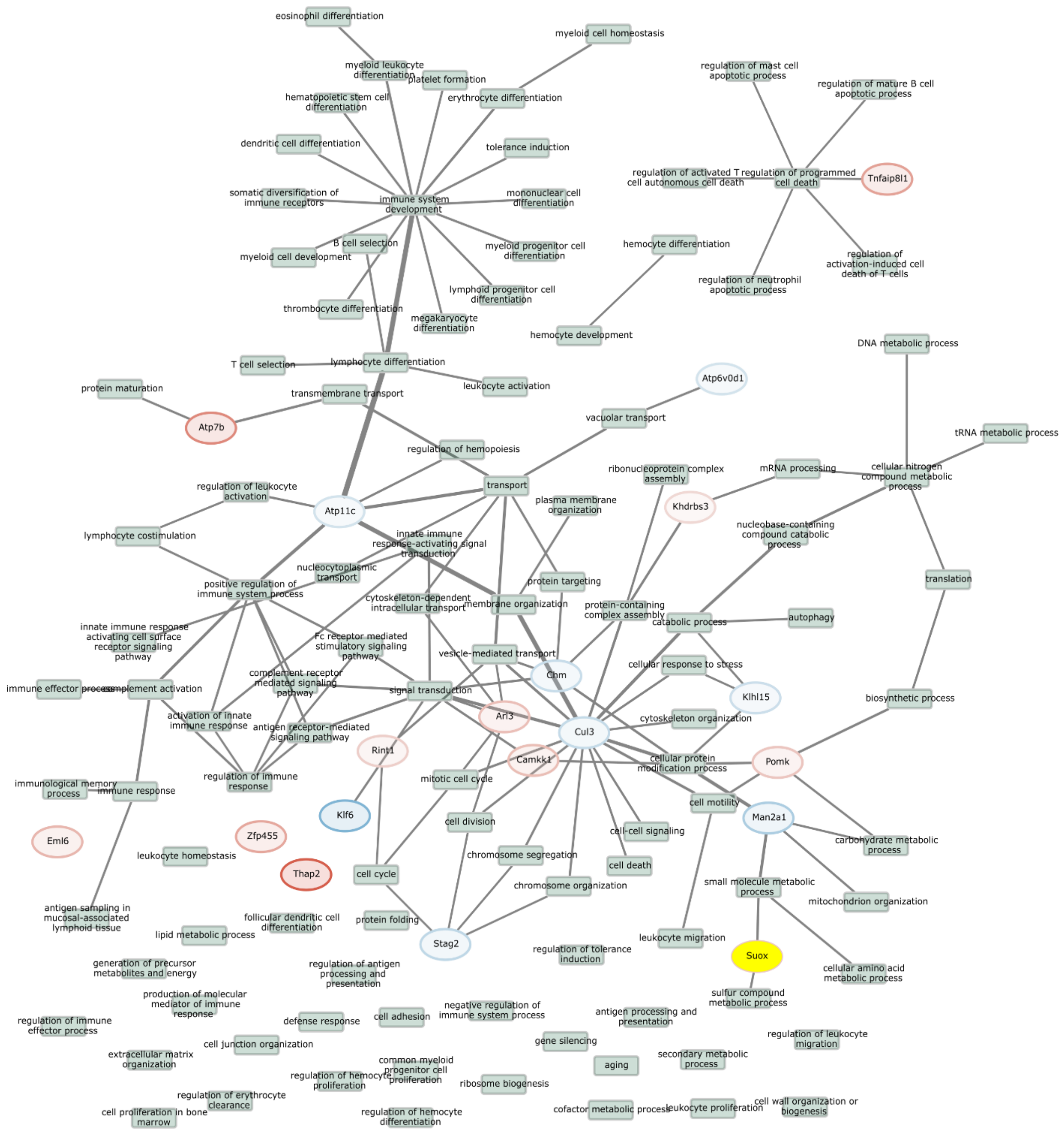

Figure S4: GOnet database gene ontology network of nineteen genes in various functional categories.

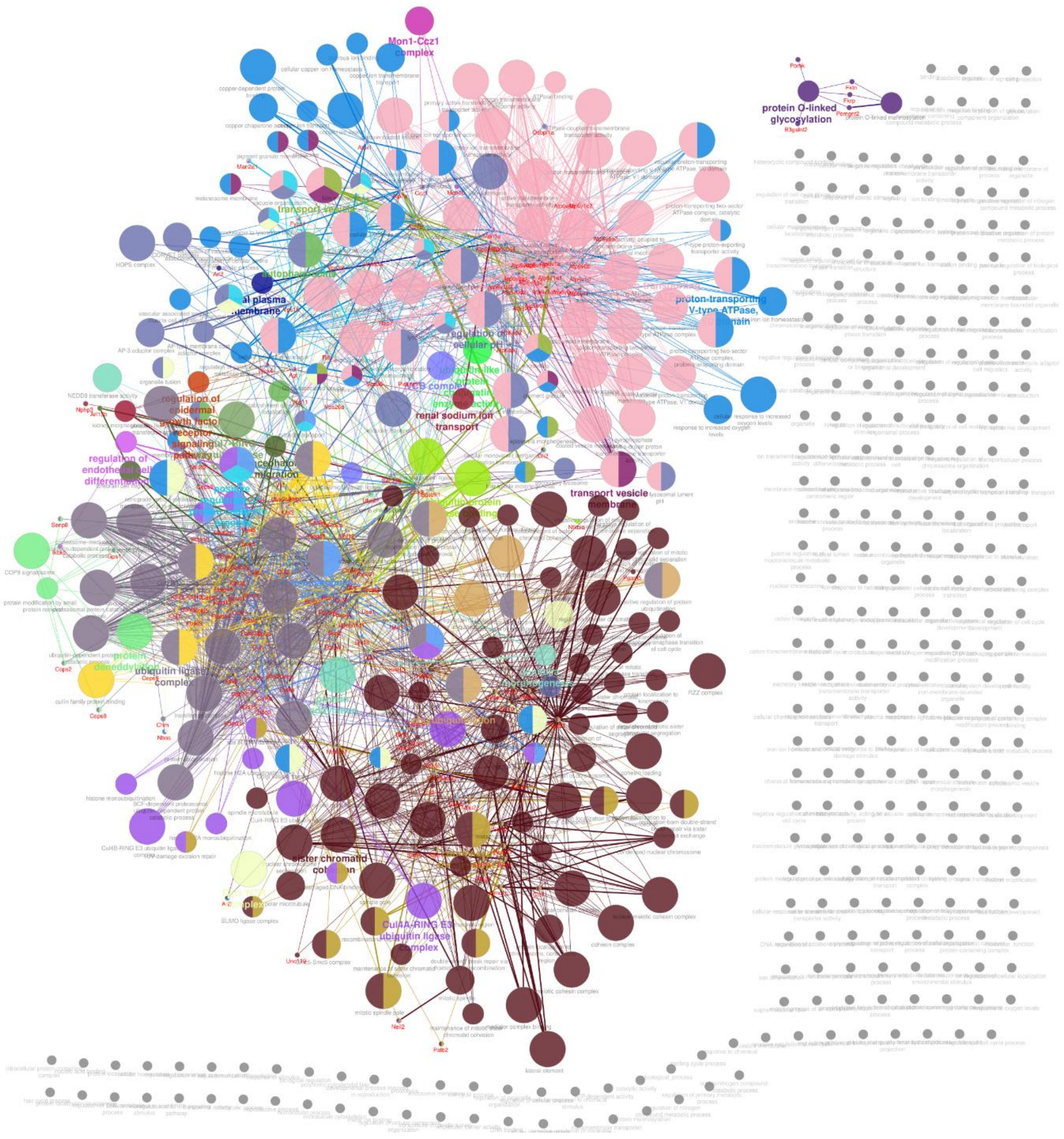

Figure S5: Gene ontology Cluego enrichment for the co-expressed Protein-Protein I interactions (PPI).
